# The Effects of Living Arrangements on Depression and Life Satisfaction in Rural Chinese Older Adults: A 20-Year Study

**DOI:** 10.1093/geroni/igaf122.3310

**Published:** 2025-12-31

**Authors:** Jin Guo, Andrew Wister, Barbara Mitchell, Shuzhuo Li

**Affiliations:** Xi’an Jiaotong University, Xi’an, Shaanxi, China (People’s Republic); Simon Fraser University, Vancouver, British Columbia, Canada; Simon Fraser University, Vancouver, British Columbia, Canada; Xi’an Jiaotong University, Xi’an, Shaanxi, China (People’s Republic)

## Abstract

**Objectives:**

China has experienced rapid socio-economic transformations over the past two decades. Based on individualization and intergenerational solidarity theories, this study explores changes in the associations between the living arrangement on psychological well-being over time and across different chronic conditions.

**Methods:**

Eight waves of longitudinal data (2001–2021) collected in Anhui, China were employed, including. 9,765 (person-year) observations. Mixed linear models containing interaction terms for time and chronic conditions were used to examine the effects of living arrangements on life satisfaction and depression.

**Results:**

Compared to living with children, the negative correlation with life satisfaction and the positive correlation with depression for those living alone or with spouse only decreased over time, while the negative correlation between living in skipped-generation household and life satisfaction also diminished. As the number of chronic conditions increases, life satisfaction declines further for older adults living alone, with a spouse only, or in a skipped-generation household. Living alone leads to a higher increase in depression, but living with others reduces it.

**Discussion:**

This study is the first to identify the changes in dynamic relationships between living arrangements on psychological well-being of older adults across time and chronic health conditions, reflecting shifts in filial piety and family culture. While older adults increasingly accept independent living, coresiding with children remains beneficial during multimorbidity. These findings contribute to the understanding of ageing in the context of economic and cultural transitions in developing regions.

